# Magnetic resonance imaging signs of high intraventricular pressure - comparison of findings in dogs with clinically relevant internal hydrocephalus and asymptomatic dogs with ventriculomegaly

**DOI:** 10.1186/s12917-015-0479-5

**Published:** 2015-08-01

**Authors:** Steffi Laubner, Nele Ondreka, Klaus Failing, Martin Kramer, Martin J. Schmidt

**Affiliations:** Department of Veterinary Clinical Sciences, Small Animal Clinic, Justus-Liebig-University, Frankfurter Straße 108, Giessen, 35392 Germany; Department of Biomathematics, Justus-Liebig-University, Frankfurter Straße 95, Giessen, 35392 Germany

**Keywords:** Hydrocephalus, Ventriculomegaly, Dog, Intraventricular pressure, Brain malformation

## Abstract

**Background:**

Magnetic resonance imaging (MRI) findings of canine brains with enlarged ventricles in asymptomatic dogs were compared to those in dogs with clinically relevant internal hydrocephalus, in order to determine the imaging findings indicative of a relevant increase in intraventricular pressure. Discrimination between clinically relevant hydrocephalus and ventriculomegaly based on MRI findings has not been established yet and is anything but trivial because of the wide variation in ventricular size in different dog breeds and individuals. The MRI scans of the brains of 67 dogs of various breeds, skull conformation and weight were reviewed retrospectively. Based on clinical and imaging findings, the dogs were divided into three groups: a normal group (*n* = 20), a group with clinically silent ventriculomegaly (*n* = 25) and a group with severe clinically relevant internal hydrocephalus (*n* = 22). In addition to the ventricle/brain-index, a number of potential subjective signs of increased intraventricular pressure were recorded and compared between the groups.

**Results:**

The ventricle/brain-index was significantly higher in dogs with relevant hydrocephalus (*p* < 0.001) and a threshold value of 0.6 was specified as a discriminator between internal hydrocephalus and ventriculomegaly. Other MR imaging findings associated with clinically relevant hydrocephalus were an elevation of the corpus callosum (*p* < 0.01), dorsoventral flattening of the interthalamic adhesion (*p* < 0.0001), periventricular edema (*p* < 0.0001), dilation of the olfactory recesses (*p* < 0.0001), thinning of the cortical sulci (*p* < 0.0001) and/or the subarachnoid space (*p* < 0.0027) and disruption of the internal capsule adjacent to the caudate nucleus (*p* < 0.0001).

**Conclusion:**

A combination of the abovementioned criteria may support a diagnosis of hydrocephalus that requires treatment.

**Electronic supplementary material:**

The online version of this article (doi:10.1186/s12917-015-0479-5) contains supplementary material, which is available to authorized users.

## Background

One of the ongoing challenges in veterinary neuroradiology is to differentiate clinically relevant hydrocephalus from ventricular enlargement in dogs. In fact, large ventricles are a common incidental finding in brachycephalic dog breeds [1–4] and have been referred to as “ventriculomegaly” to differentiate this finding from relevant internal hydrocephalus. These dogs are considered to be asymptomatic and are not thought to have associated increased intraventricular pressure (IVP) [5–7]. However, there is no threshold level of ventricular volume that discriminates the two conditions [8–11]. Assessment of ventricular size alone is therefore not helpful to evaluate whether neurological signs are a potential consequence of brain damage due to an existing high IVP and ventricular dilation. This is of particular importance because inflammatory/infectious brain disease, which might be present in addition to ventricular enlargement and may lack other specific imaging findings, can remain undetected. Hence, secondary ventriculomegaly is thought to sometimes be misdiagnosed as relevant internal hydrocephalus and interpreted to be the cause of clinical signs in dogs affected by inflammatory/infectious disorders.

Magnetic resonance imaging (MRI) is the method of choice for the assessment of hydrocephalus in humans [12, 13] and animals [14] and has been used as the primary means of diagnosis for internal hydrocephalus in our institution. Detailed morphological abnormalities indicative of increased IVP beyond ventricular dilation may be identified by means of MRI [15, 16]. The aim of this study was to determine the morphological and morphometric findings indicating a high IVP by documenting their presence in MRI studies of dogs with symptomatic internal hydrocephalus in contrast to dogs with asymptomatic ventriculomegaly and normal dogs.

## Methods

### Animals

The archive of MRI scans at the Justus Liebig University (JLU), Germany, was retrospectively searched for MR image reports including the diagnoses “within normal limits”, “internal hydrocephalus“and “ventriculomegaly” or “enlarged ventricles”. MRI reports for each series were reviewed by board-certified radiologists. MR imaging had to include sagittal, transverse and dorsal scans of the entire brain. The sex, age and body weight of the dogs at the time of scanning were recorded.

Subjects were divided into the following groups. Group 1 included 20 dogs, whose brain and ventricles had been determined to be “within normal limits”. Group 2 included 25 dogs, in which a distension of the lateral cerebral ventricles had been noted as an incidental finding. The presence of ventriculomegaly was based on the following criteria. The majority of dogs have very narrow and slit-like horns of the lateral ventricles. In the finding of large ventricles/ventriculomegaly, the interpreter subjectively noted a greater proportion of the intracranial volume occupied by the lateral ventricles. The closely spaced walls of the temporal horns and/or the olfactory recesses were separated by cerebrospinal fluid (CSF) in these brains and the lacking of a septum pellucidum created a large connection between the first and second ventricle [17]. Dogs in Groups 1 and 2 were examined for diseases not primarily related to the brain, as e.g. intraorbital inflammation, facial nerve paralysis, middle ear disease, etc., or seizures. None of these dogs showed signs of parenchymal changes of the brain. Group 3 included 22 dogs with internal hydrocephalus and clinical signs of forebrain disease that subsided after implantation of a ventriculoperitoneal shunt.

Approval from the ethics committee of the Justus-Liebig-University was not sought as retrospective studies of images stored in the archive are not subject to ethical review.

### Imaging technique

Imaging was performed using a 1.0 Tesla MRI scanner (Phillips Intera Gyroscan, Phillips Healthcare, Hamburg, Germany). Images included sagittal, transverse and dorsal T2-weighted (Turbo Spin Echo, TR: 1900, TE: 108, slice thickness 3 mm) and transverse fluid-attenuated inversion recovery (FLAIR) sequences with three-dimensional Fast Field Echo (FFE) T1-weighted pre- and post-contrast medium administration (TR: 588, TE: 15, slice thickness 1 mm).

### Image analysis

Imaging features indicating a high IVP were reviewed referring to human studies. Furthermore, gross and histopathological examinations of the brain of dogs with naturally occurring as well as experimentally induced hydrocephalus yielded characteristic findings assigned to increased IVP identifiable on MRI scans [18–20]. One PhD student and a board certified neurologist reviewed the MRI studies for the presence of findings associated with internal hydrocephalus for the study groups and controls. The experiments were performed using anonymized and randomized image data sets. The observers were blinded to the breed and diagnosis of the individual dogs. All of the following measurements/interpretations were made by the two observers independently to determine interobserver variability.

### Morphological criteria

1. Expansion of the third ventricle represented by flattening of the interthalamic adhesion and a diminished suprasellar cistern [3, 21]. A deformation of the interthalamic adhesion was assumed when it was not distinctly circular on midsagittal plane images (Fig. 1e). Narrowing of the suprasellar cistern was diagnosed in transversal images based on the lateral bulging contours of the hypothalamus diminishing the CSF of the adjacent cistern (Fig. 1b/c ).

**Fig. 1:**
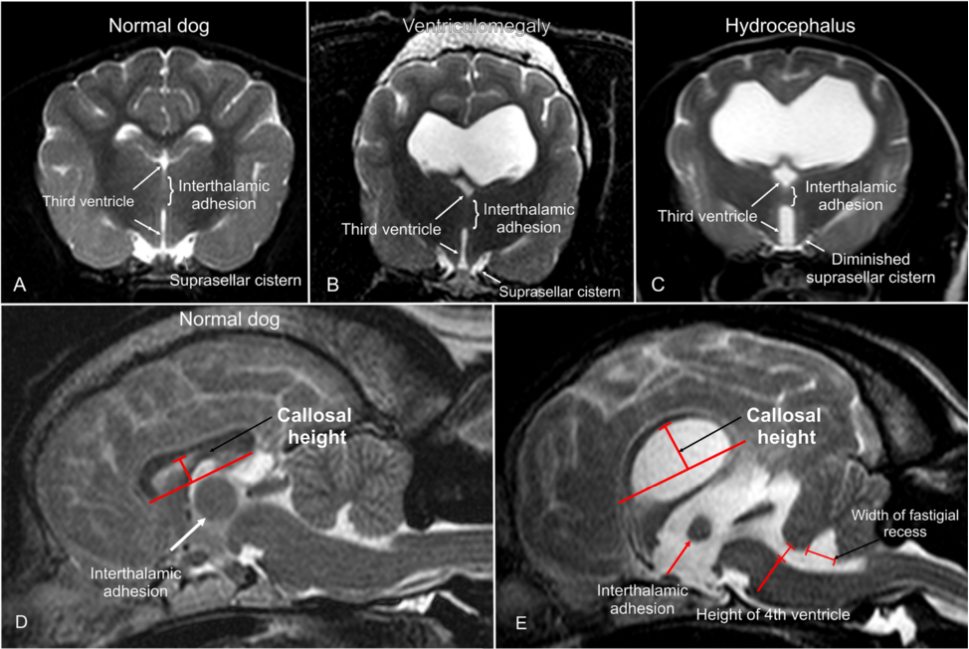
MRI-signs of increased intraventricular pressure. Transverse (**a**–**c**) and sagittal (**d**, **e**) T2-weighted MR-images of a normal dog brain (**a**, **d**), a dog with ventriculomegaly (**b**, **e**), and with internal hydrocephalus (**c**). The finding of an expanded third ventricle and the measurement of the corpus callosum height (callosal height) and dimensions of the fourth ventricle is demonstrated
2. Disruption of the internal capsule adjacent to the caudate nucleus [18].
3. We assessed T2-weighted dorsal images for the presence of unilateral or bilateral disruption adjacent to the caudolateral pole of the caudate nucleus, leading to separation of this structure from the internal capsule (Fig. 2b).

**Fig. 2:**
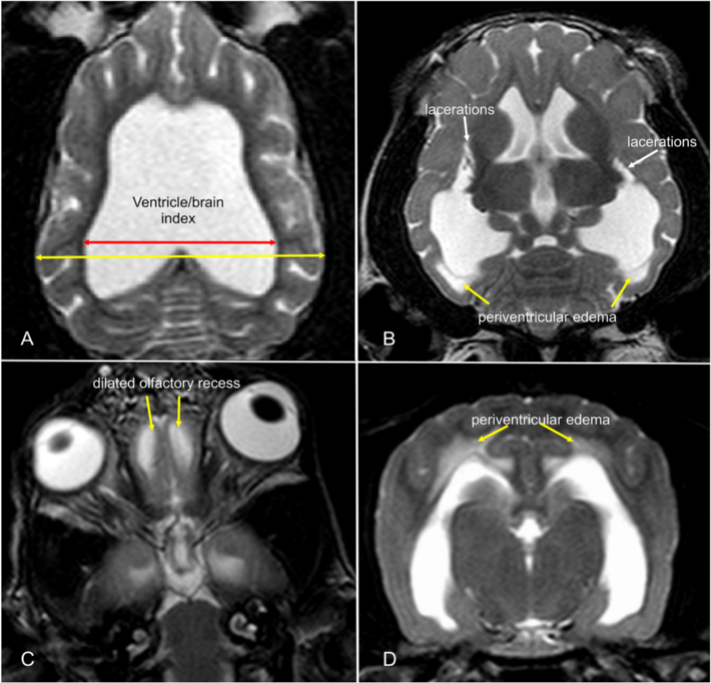
MRI-signs of increased intraventricular pressure. Dorsal (**a**–**c**) and transverse (**d**) MR-images of dogs with internal hydrocephalus showing signs of increased intraventricular pressure (IVP). The amount of distension is measured by the ventricle/brain-index (**a**). The IVP leads do dilation of the olfactory recesses (**c**). Periventricular edema occurs if the intraventricular pressure exerts the compliance of the brain parenchyma (**d**). This can also lead to lacerations of the white matter adjacent to the caudate nucleus (**b**)
4. Periventricular edema (PVE) [22–26]. Dorsal and transverse T2-weighted images were reviewed for the presence of high signal intensity within the periventricular white matter complemented by simultaneous hypointensity in T1-weighted sequences and hyperintensity in FLAIR (Fig. 2b/d).
5. Narrowing of cerebral sulci and obliteration of the subarachnoid space around the dorsal convexity of the cerebral hemispheres [13, 20]. The absence of a hyperintense subarachnoid space and/or the presence of narrowed cortical sulci were recorded on transverse T2-weighted images at the level of the interthalamic adhesion (Fig. 3c).

**Fig. 3:**
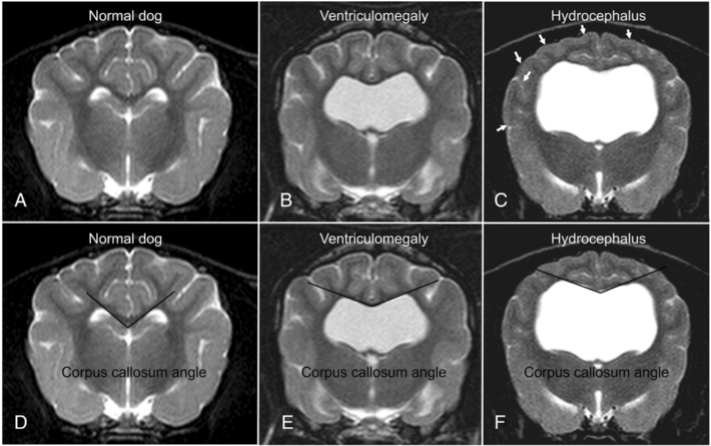
MRI-signs of increased intraventricular pressure. Transverse MR-images of a normal dog (**a**, **d**) and a dog with ventriculomegaly (**b**, **e**) and internal hydrocephalus (**c**, **f**). The finding of a compressed subarachnoid space (*white arrows*, **c**) and the measurement of the corpus callosum angle is demonstrated
6. Dilation of the olfactory recess(es) [15, 20, 27]. Transverse and dorsal T2-weighted images were evaluated for the presence of a hyperintense signal (CSF) within the olfactory bulb continuous with the frontal horns of the lateral ventricles (Fig. 2c).
7. Presence of cerebellar deviation [19, 28, 29]. A cerebellar deviation was considered when the cerebellum protruded to the level of or through the foramen magnum [30].

### Morphometric criteria

1. Corpus callosum angle (callosal angle) [12, 16, 31]. The callosal angle describes the angle between the corpus callosum as the center and the dorsomedial internal surfaces of the lateral ventricles as angle legs on a transverse MR image. We determined it in analogy to human patients on T2-weighted images at the level of the pituitary gland (Fig. 3d-f).
2. Corpus callosum height (callosal height) [32–34]. The elevation of the corpus callosum was measured in the midsagittal image using a straight line connecting the splenium and rostrum (callosal line [33]) and a perpendicular line to the dorsal-most extension of the body of the corpus callosum (Fig. 1d/e).
3. Ventricle/brain (VB)-index. The ventricle/brain index was evaluated on dorsal T2 images. The VB-index was defined as the maximum continuous distance between the internal borders of the ventricles divided by the maximum width of the brain parenchyma in the same image (Fig. 2a).
4. Expansion of the fourth ventricle [20, 35, 36]. In normal dogs, the cerebellum is in contact with the medulla in midsagittal images. The height of the fourth ventricle was determined on a T2-weighted midsagittal plane image at its widest extension in the dorso-ventral direction. Additionally, the width of the fastigial recess was determined at its widest extension in the rostro-caudal direction (Fig. 1e).

### Statistical analysis

All statistical analyses were performed using the statistical software package BMDP [37]. With respect to Groups 2 and 3, for each of the qualitative criteria, the statistical significance of the differences between these groups was assessed by considering the two-way frequency table and by performing the Chi-square test for homogeneity or Fisher’s exact test depending of the size of the smallest expected value in the table. These tests were not performed incorporating the normal group since, by definition, these criteria do not occur.

For the quantitative criteria, the linear relationship to body weight was verified by regression analysis and scatterplots. The deviation from the normal distribution was checked using the normal probability plot of the model residuals (Q-Q-plot) for each variable. Subsequently, a one-way analysis of covariance was performed for each of the individual criteria to include the influence of the body mass and the groups’ influence on the means of the measured quantities simultaneously. Variables with a dependence on body weight were calculated for the adjusted group means. If there were global significant differences in the mean values between the groups, a pairwise comparison of group means by the Student-Newman-Keuls test was performed. The parameters expansion of the fourth ventricle and callosal height were logarithmically transformed throughout the analysis because the distribution of their values was skewed to the right. In all statistical test procedures, a significance level of *p* = 0.05 was used.

The V/B index was only calculated for Groups 2 and 3. As a dependence on body weight was not present for the V/B-index, the group comparison was performed using a simple *t*-test for independent samples. For the estimation of a threshold value, a parametric reference interval calculation was subsequently performed [38]. In addition, the upper 95 % confidence margin was determined for Group 2. Receiver operating characteristic (ROC) analysis was performed in order to optimize the selection of the cut-off value to achieve maximal sensitivity and specificity.

The precision of the interrater variability of the quantitative findings was determined using Bland-Altman analysis to compare the differences between the first and second measurements of each dog. The differences between the two measurements were then plotted against the average (mean) of the two measurements. Good reproducibility was assumed when 95 % of the differences were within two standard deviations. Interrater variability of the qualitative findings was assessed using kappa statistics.

## Results

### Animals

Information regarding the breed, bodyweight, age, gender and indication for MRI/final diagnosis of all dogs are summarized in Additional file 1.

### Analysis of morphological criteria

All qualitative criteria were found to differ significantly between Groups 2 and 3, except for cerebellar deviation. Expansion of the third ventricle, as represented by deformation of the interthalamic adhesion, was significantly more frequent in dogs with a relevant hydrocephalus (*p* < 0.0001). The same applies for the presence of periventricular edema (*p* < 0.0001) and dilation of the olfactory recess(es) (*p* < 0.0001), both of which were only present in dogs with hydrocephalus. Thinning of the cerebral sulci (*p* < 0.0001) and thinning of the subarachnoid space (*p* = 0.0027) as well as disruptive lesions of the internal capsule adjacent to the caudate nucleus (*p* < 0.0001) were also found exclusively in the hydrocephalic group. A kappa value of one revealed excellent interobserver agreement.

### Analysis of the quantitative criteria

The mean V/B-index was 0.54 (range: 0.44–0.65) in the dogs with ventriculomegaly (Group 2) and 0.73 (range 0.58–0.92) in the dogs with hydrocephalus (Group 3), which represents a significant difference between these groups (*p* < 0.001). The upper 95 % reference limit for the V/B-index between ventriculomegaly and hydrocephalus was calculated to be 0.62. In order to further optimize sensitivity and specificity, ROC analysis was performed and an optimal cut-off value of 0.605 was calculated. At a cut-off value of 0.605 for sensitivity and a specificity of 92 % were determined.

The callosal height in group one (normal dogs) ranged from 3.7 mm to 7.3 mm with a mean of 5.2 mm. In Group 2 (ventriculomegaly), it ranged from 5.0 mm to 13.7 mm (mean 8.7), and in Group 3 (hydrocephalus) from 6.6 mm to 23.5 mm (mean 12.6 mm). The mean values were significantly different between all groups (*p* < 0.01).

ANCOVA revealed a significant influence of body weight on this value (*p* = 0.001). Therefore, the values were related to the adjusted means of the body weight of all groups. Differences between all groups remained significant. Adjusted means of callosal height were significantly higher in dogs with ventriculomegaly than in normal dogs (*p* < 0.01) and significantly higher in hydrocephalus dogs compared to the ventriculomegaly group (*p* < 0.01). The 95 % reference limits for callosal height can be calculated for dogs with different body weights using the following equation:

$$ 10.2 \times {10}^{.\mathrm{0.006} \times \mathrm{B}\mathrm{W}} $$

No statistically significant difference could be detected between dogs with hydrocephalus (Group 3) and those with enlarged ventricles (Group 2) regarding the corpus callosum angle (*p* = 0.961). However, both groups differed significantly from the normal group (*p* < 0.01). Body weight did not affect this measurement value.

Expansion of the fourth ventricle in normal dogs differed significantly from dogs with ventriculomegaly (*p* < 0.05) and dogs with hydrocephalus (*p* < 0.01), but there was no significant difference between the ventriculomegaly and the hydrocephalus group (*p* = 0.842). Body mass is suspicious to affect this measurement value (*p* = 0.064).

Width of the fastigial recess revealed no significant difference between dogs of each group (*p* = 0.879). Body weight did not affect this measurement value.

**Fig. 4:**
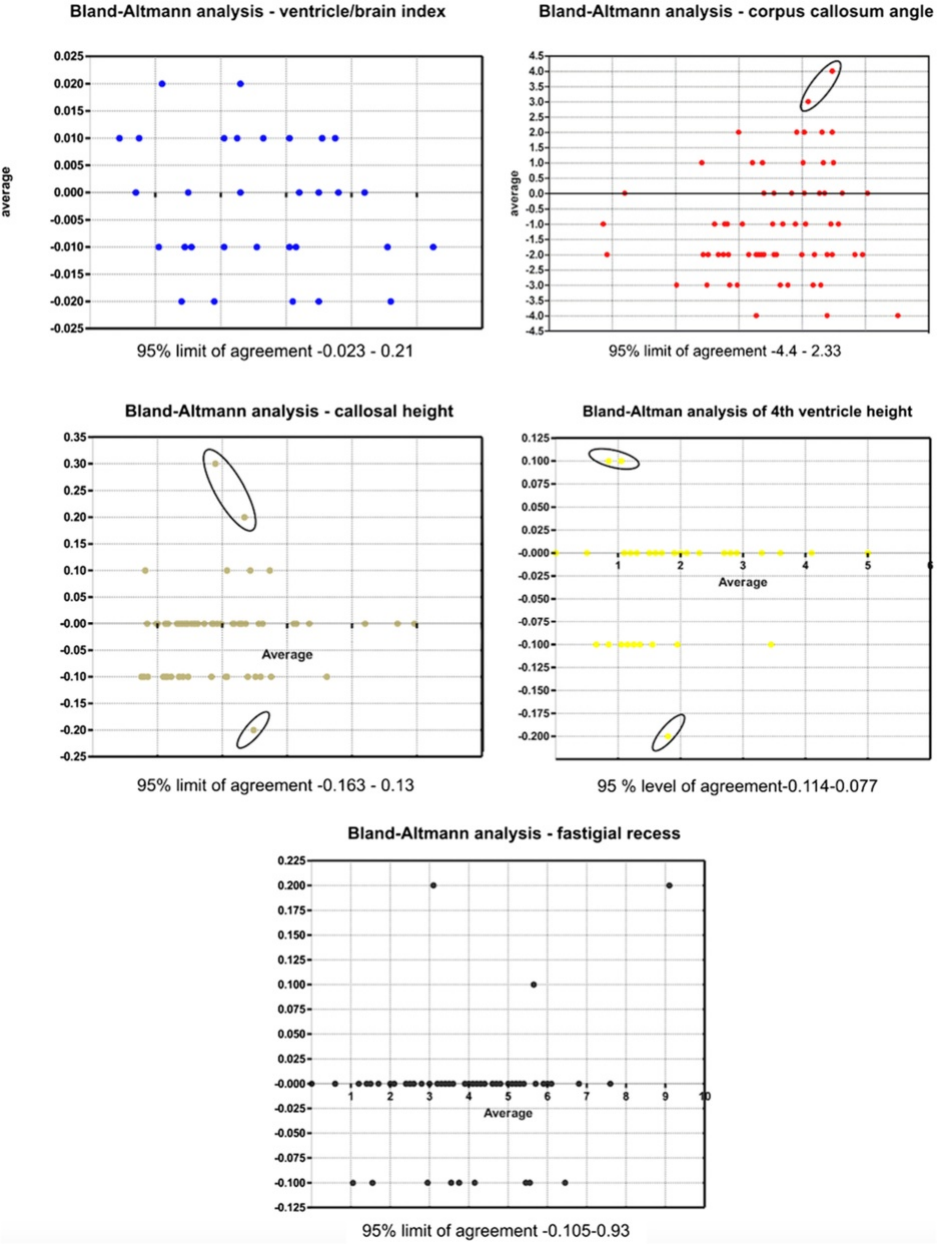
Repeatability analysis. Graphical presentation of the comparison of two measurements of the quantitative measurements in Bland-Altman plots. The differences between the two measurements are plotted against the averages of the differences. Ninety five percent of all differences are within two standard deviations representing an excellent repeatability. Outliers are marked with a black rim

The assessment of repeatability is shown in Fig. 4. Ninety-five percent of the differences between the first and second measurement are less than ± 2 standard deviations (SD’s) from the mean difference. The Bland-Altmann analysis revealed significant agreement between the raters.

## Discussion

In certain clinical situations, the diagnosis of hydrocephalus may be difficult to establish, particularly in small brachycephalic dog breeds that tend to have relatively larger ventricles in comparison to mesaticephalic dogs [8, 39]. Ventricular enlargement may also be characteristic of other neurodegenerative diseases and normal aging in dogs [40]. Mere determination of the ventricular volume was previously not successful in identifying clinically relevant hydrocephalus [7, 8]. The identification of clinically relevant ventricular distension is extremely important for the indication for CSF shunting. We have seen a number of dogs with neurological signs, which have been referred to our hospital for ventriculo-peritoneal shunting, in which a diagnosis of internal hydrocephalus was made based on the finding of ventricular enlargement alone. CSF examination, however, often revealed idiopathic inflammatory diseases (necrotizing encephalitides), which primarily require medical treatment. Inflammatory brain diseases may easily be overlooked, and large ventricles can be easily misinterpreted as the underlying cause for present neurological signs in those dogs. Therefore, we aimed to identify further characteristic morphological changes using MRI studies of the dog’s brain related to an assumed increase in intraventricular pressure. Our results show that morphological differences exist between hydrocephalus and ventriculomegaly, which might be useful in the differentiation of these two entities.

The findings in the hydrocephalus group indicate parenchymal changes based on pressure forces on the parenchyma. The gradual expansion of the ventricular system follows a predetermined sequence, starting with the temporal horn of the lateral ventricles. Subsequently, the remainder of the lateral ventricles expands, followed by the fourth ventricle [20]. The third ventricle is the last one to show distension. It has been proposed that periventricular white matter of the ventricles is exposed to expansive stress, especially in the region of the ventricular horns. At the same time, expansion of the ventricles leads to compressive forces on the thalamus, rendering high pressures mandatory in order to expand the thalamus that surrounds the third ventricle [41]. In human neuroradiology, dilation of the third ventricle has been shown to indicate a high pressure gradient between the ventricle and the ventral subarachnoid spaces (interpeduncular cistern and hypophyseal cistern). Such a finding serves as a major indication for third ventriculostomy in children [42] and might also prove useful for canine patients in the future.

There are many ways of measuring relative ventricular size in human medicine, using linear ratios, area and volumetric measurements [43]. The most common measurement is the Evans' index that describes the ratio of the transverse diameter of the anterior horns (rostral horns in dogs) of the lateral ventricles to the largest diameter of the brain in humans. Evans’ index has also been used to measure the ratio between the lateral ventricles and brain parenchyma in hydrocephalic dogs. However, it has been found to underestimate the degree of ventricular distension [44]. Other measurements estimating the relation between frontal horn diameter and brain parenchyma have been used on different levels and image planes in the dog brain using different imaging modalities [7, 9, 44–49]. Only one study found a significant difference between dogs with ventriculomegaly and hydrocephalus [46]. As mentioned above, experimental studies have shown that the temporal horns of the ventricles dilate first in dogs, followed by other parts of the ventricle [20]. This was also reported in children [50] and in mathematical models of hydrocephalus using finite element analysis [41]. The maximum continuous extension from one ventricle to the other was measured in temporal horns in our study rather than classical measurement of the frontal horns based on Evans’ method, which may explain the significant results of our study in contrast to those of previous studies. Further studies with three-dimensional-morphometric measurements are needed to determine whether our VB-index might underestimate ventricular distension as well.

Platt and Garosi [15] described dilation of the olfactory recess to be suggestive of increased IVP. Dilation of the olfactory bulb cavity was also noted in experimentally induced hydrocephalus [20, 28]. It was interpreted as transmission of high pressure from the frontal horns to the normally non-expanded recesses that occurs only in late stages of increased IVP.

Changes in the appearance of the corpus callosum have been reported in human patients with hydrocephalus. Structural changes due to high intraventricular pressure include stretching and upward displacement of the body of the corpus callosum and concurrent downward depression of the fornix in humans [33–35]. This was also found in dogs with experimental hydrocephalus [20]. Demyelination of the callosal axons has been suggested to be the underlying cause of increased compliance of the commissural fibers, leading to the upward bowing of the corpus callosum with increased pressure [49]. Other than the parenchyma surrounding the lateral ventricles, the corpus callosum is situated beneath the rigid falx. The connective tissue of this structure exerts additional resistance against the expanding ventricle and higher pressures are needed to elevate the corpus callosum.

The corpus callosum angle reflects dorsal distension of the dilated lateral ventricles [16]. In human medicine, a callosal angle of less than 90 ° is a criterion for the identification of high intraventricular pressure [12]. Virhammar et al. [31] showed that patients with a smaller callosal angle are more likely to respond to shunting, both clinically and with reconstitution of the cerebral parenchyma. To our knowledge, the callosal angle has not been measured in canine patients before. There were no significant differences between the callosal angles of dogs with ventriculomegaly and those with hydrocephalus. This might be related to the high callosal height in the hydrocephalus group. Moving the center of the angle (i.e. the corpus callosum) upwards automatically leads to a wider angle in these dogs, which can explain the lack of difference between the groups.

Focal periventricular white matter edema has been attributed to the transependymal absorption of cerebrospinal fluid that follows the pressure gradient from the ventricle to the parenchyma [51–53]. Another interpretation describes PVE as areas with restricted absorption capacities for extracellular water that flows from the parenchyma towards the ventricles [54]. This has been found in acute and subacute phases of human hydrocephalus [55], experimental hydrocephalus in dogs [22–24] as well as in idiopathic hydrocephalus [26]. Water diffusion within periventricular white matter has been found to be highly dependent on age in children and the diffusion capacity dramatically decreases with normal brain maturation [56]. Although the median age is lower in the hydrocephalus group, both groups include immature and mature dogs, which is why early age does not seem to be a prerequisite for the occurrence of PVE.

Expansion of the ventricles can cause compression of the cortical subarachnoid CSF space [53, 57], also leading to decreased space within the sulci. This finding has been seen in hydrocephalic humans [13] and animals [20]. High pressure in the ventricles and low pressure in the subarachnoid space (SAC) are prerequisites for a constricted CSF space. The pressure from the ventricular system has been reported to not be transmitted homogeneously over the entire surface of the brain hemispheres, but primarily into the dorsolateral direction [58, 59]. Due to this fact, we examined the SAC and the sulci on the cerebral convexity on the level of the interthalamic adhesion for consistency.

A disrupted ependymal lining and formation of false diverticula have been found in human patients with severe hydrocephalus [54], as well as in dogs [60, 61]. Stretching and eventual destruction of the internal capsule adjacent to the caudate nucleus was observed as a consequence of overpressure in experimental [20] and naturally occurring hydrocephalus in dogs [18].

The determination of the presented qualitative changes of the brain parenchyma should be assumed to be a subjective method and may be less accurate than quantitative methods. However, we were able to show that simple visual evaluation has a high interrater agreement.

In dogs, skull and brain size and morphology differ between brachycephalic and mesaticephalic breeds. In brachycephalic dogs, growth reduction of the skull base has been documented [62], which has a potential influence on the morphology of the brain stem and the measured distances in this area as well. Breed-specific values are necessary to use the same parameters as in humans. For this reason, we decided to confine our analysis to a simple assessment of the third ventricle based on the shape of the interthalamic adhesion. A deformation of the interthalamic adhesion has been identified in dogs with high intraventricular pressure and was also interpreted as a sign of third ventricular distention due to overpressure [3, 20, 21].

The distension of the cerebral ventricles and with it the whole forebrain can lead to caudal deviation of other brain regions [63]. Deviation of the cerebellum through the foramen magnum has been described in the development of experimental hydrocephalus [19, 28, 29] as well as in naturally occurring hydrocephalus [64]. However, the impression of cerebellar deviation can be found in a number of small brachycephalic dog breeds, even with normal ventricles, which explain the lack of significance of this finding. Enlargement of the fourth ventricle has been described in human hydrocephalus [55], and fourth ventricle dimensions are significantly larger in human patients with intraventricular overpressure. Dilation of the fourth ventricle has also been observed in animals with experimental communicating hydrocephalus [20, 35, 36]. The reasons behind the lack of a difference between the groups concerning this finding remain undetermined.

It is widely assumed that elevated pressure is a direct result of an impaired CSF flow from the ventricles to its point of absorption [54, 57, 63, 65]. This leads to a progressive destruction of white matter in dogs as a consequence of high IVP, which has been thought to not be present in ventriculomegaly. However both dogs with ventriculomegaly and hydrocephalus were significantly different from normal dogs for most of the quantitative MRI findings that we examined. Based on these rather gradual than principal differences, it would be unsound to assume that ventriculomegaly is not a consequence of overpressure. Long-term studies measuring IVP in dogs with dilated ventricles are needed. Accumulation of CSF and distension of the ventricles might occur very slowly in dogs with ventriculomegaly, giving the brain time to adapt to changes in periventricular perfusion and other pathological changes. It is reasonable to expect that the degree of intraventricular pressure determines the speed and severity of parenchymal injury and thereby functional brain deficits. Moderately increased pressure or intermittently high intraventricular CSF pressure might produce temporary phases of ischemia in the periventricular white matter. The chronic cumulative effects of these ischemic events could produce very slowly progressing periventricular tissue atrophy. Although raised IVP might cause slowly progressing tissue damage, this does not necessarily produce clinical signs. Signs of dementia, such as those found in humans, might also pass unnoticed in a classical neurological examination. The clinical use of the identified imaging findings must be judicious. Although some parameters revealed a clear cut-off, it is likely that the combination of all these measurements may yield a better evaluation about high intraventricular pressure using morphological MRI. A more detailed classification of canine hydrocephalus and the clear discrimination of the underlying causes are required to confirm their usefulness in each individual dog.

## Conclusion

A V/B-index greater than 0.6 together with an elevated corpus callosum and the presence of a deformed intermediate mass, periventricular edema, dilation of the olfactory recess, thinning of the sulci and/or the subarachnoid space or disruption of the internal capsule adjacent to the caudate nucleus are highly suspicious for clinically relevant dilation of the lateral cerebral ventricles due to increased IVP.

## Additional file

Additional file 1:
**Breed, bodyweight, age, gender and indication for MRI/final diagnosis of the examined dogs in the different groups.**

## Abbreviations

ANCOVA: Analysis of covariance
CSF: Cerebrospinal fluid
FFE: Fast field echo
FLAIR: Fluid-attenuated inversion recovery
IVP: Intraventricular pressure
JLU: Justus Liebig University
MRI: Magnetic resonance imaging
PVE: Periventricular edema
ROC: Receiver operating characteristic
SAC: Subarachnoid space
SD: Standard deviation
V/B-index: Ventricle/brain-index
